# What Can Dentists Do to Advance Their Profession?

**Published:** 1852-10

**Authors:** 


### ARTICLE VII.

### *What can Dentists do to Advance their Profession?*

THAT they ought to do something, is plain enough. The profession is yet unorganized. It exists in individual representatives, scattered over this vast country, little known to each other, with no central bond of union, and pursuing no plan for common benefit. By their individual exertions, much has been done; and by the little attempts which have been made towards combination, what individuals have done has been seen and examined, and very partially communicated to the fraternity—professional feeling has been created in the breasts of some, and diffused, in more or less intensity, throughout the multitude—dentistry has got to itself a name. It is now a distinct thing. It is talked about, and written about, and thought about, somewhat. It is no longer an accident of one's life to be a dentist. Dentistry is a vocation, like other vocations—to be chosen by the young, pursued by the middle aged, and approvingly nodded over by the old; it has taken its place, decidedly, among the doings of human life.

So much has been accomplished—not without long and patient effort;—and those who have accomplished it, are worthy of everlasting remembrance.

But much remains to be done. The work is yet only begun. Unless it be zealously prosecuted, it will come to naught.

What, then, is to be done?

Let us see what is doing. There is a national convention of dentists, called the American Society of Dental Surgeons. This body holds an annual meeting, attended by, perhaps, a score of members. Its transactions are interesting; and the effect of the meeting upon those who attend them, is felt in the cultivation of social feeling and the incitement to professional effort.

If this body could be, indeed, made national, it would be exceedingly useful. It has done much to elevate the dignity of the profession; but, latterly, its influence seems to be less felt, and to have become more circumscribed.

There are several local societies, all small in numbers; and though the members are able and zealous, these associations are almost impotent for extensive good.

There are several journals devoted to the discussion of dental subjects. Some of these are conducted with spirit; all, as far as we know, are slenderly supported.

There are several colleges chartered for the education of dentists. Some of these are of late creation, and, as yet, of course, have done little or nothing towards the great purpose for which they were designed. Unfortunately, none of these schools have ever been properly supported by dentists at large; and even among those, from whom liberal feelings towards these institutions might have been expected, a paltry envy or jealousy has been permitted to govern the conduct of more or less.

Lately, one or two lectureships on dentistry have been appended to the faculties of medical schools, which will do about as much for the dental students as lectureships on navigation.

Such is the present condition of things. What can be done to amend it?

In the first place, then, we would suggest that in every county, town, or city, where there are five dentists, a society be formed, for mutual improvement and the advancement of the profession. We would exclude from these primary association, no practicing dentist who stands fairly before the public as an honest and correct man. There are hundreds, perhaps thousands of such, who are but poor dentists. They have been very imperfectly educated, and may even be ignorant of their own ignorance. Yet, many of them would gladly improve, if they could, and few of them would not be inspired with some pride in the profession, if brought into collision with the better minds, who would naturally and necessarily take the lead in these societies. Many a clumsy operator would be made ashamed of his clumsiness, and many an ignoramus made ashamed of his ignorance; and many a man, who, while protected by his isolation, had stooped to dirty things, would shrink from dirtiness, when the eyes of a fraternity were upon him. Hence, it would follow, that many, even of these people would be improved; and, at any rate, they would cease to propagate their own kind. The society would furnish such a standard of acquisition, or direct such a course of instruction, as would prevent those men from rearing pupils in their own image.

The society, once established, might carry out its purposes in its own way. Essays might be read, addresses or lectures delivered, conversation had, and, occasionally, sociability and good feeling might be promoted, by *eating together*.

Your readers may be amused at this latter suggestion; but the history of the world shows, that no good is done in it, without much eating. Every sensible project is better digested over a good dinner, and every benevolence is promoted by intro-gastronomic courtesies. Even the missionary societies in England give breakfasts, and the temperance societies have tea parties, and the grave governors of the earth gather, and dispense wisdom at cabinet dinners. Does not the American Medical Convention eat suppers? If not, we would like to know what they do. Well, let dentists eat together, and learn not to devour each other, as they do now, but love one another, as men should, knowing that each has a stomach to be filled, and has a right to provide means to fill it, by the exercise of an honest vocation, and, therefore, should not be impeded therein by an omnivorous competitor.

These town and county associations might send delegates to form a State Society, to meet annually, and the State Societies might send delegates to form a great National Conference, to meet every three years.

All these societies might find enough to do to make their sessions interesting and instructive. It would be presumptious, as well as premature, for us to suggest the routine of exercises to be established, or the plan of operations to be pursued. If we can only get the dentists to come together, with a will to the work, they will find a way.

The great subject of education would then receive proper attention, and some definite plan of preparation for, and introduction into the profession fixed upon. That dental colleges offer the only possible means of thorough instruction to the many, is certain, and to this conviction, dentists and the community must come at last. For a time, some will adhere to the old ways. It is hard to get men of the slow coach style out of a rut in which they have been rolling and floundering for years; but they find it equally hard to persuade men of the locomotive variety, to prefer their course; and all young Americans are locomotives in breeches.

A farmer was lately taken by a city friend through his new house, replete with modern conveniences and luxurious inventions. Among other things, his attention was directed to the apparatus by which water was conveyed to all the chambers, and he was asked what he thought of it. "Why," said he, "it may all do well enough for you, city folks, but *I* would rather have a little nigger to run with a pitcher to the spring." There is no accounting for tastes, but convenience always becomes popular in the long run. If dentists would drop their pitiful jealousies of one another; if they would cease looking out of the profession for a loan of respectability, and set themselves to work, to create a respectability for itself—if, instead of watching their young colleges with censorious eyes, they would give their influence to building them up—if they would read their own journals, and if they do not like the contents, write better, and so enable the editors to improve them—if they would associate together, upon some democratic plan of organization, by which the better informed and the better known might mingle with the more humble in station and acquirements—if they would cease their attempts to tie the profession to the tail of certain medical colleges, where it will only aid in the jingle by which witless youth may be attracted—if they would do these things, they would prosper. Will they?

FAUCHARD.

